# Human tumor necrosis factor alpha affects the egg-laying dynamics and glucose metabolism of *Schistosoma mansoni* adult worms in vitro

**DOI:** 10.1186/s13071-022-05278-8

**Published:** 2022-05-24

**Authors:** Ednilson Hilário Lopes-Junior, Claudio Romero Bertevello, Gilbert de Oliveira Silveira, Camila Banca Guedes, Gratchela Dutra Rodrigues, Viviane Sousa Ribeiro, Murilo Sena Amaral, Cristina Takami Kanamura, Pedro Luiz Silva Pinto, Rodrigo Ferreira Krüger, Sergio Verjovski-Almeida, Katia Cristina Oliveira

**Affiliations:** 1 Departamento de Microbiologia, Imunologia e Parasitologia, Escola Paulista de Medicina, Universidade Federal de São Paulo, São Paulo, Brazil; 2 Laboratório de Parasitologia, Instituto Butantan, São Paulo, Brazil; 3 Departamento de Bioquímica, Instituto de Química, Universidade de São Paulo, São Paulo, Brazil; 4 Departamento de Microbiologia e Parasitologia, Instituto de Biologia, Universidade Federal de Pelotas, Pelotas, Brazil; 5 Centro de Patologia, Instituto Adolfo Lutz, São Paulo, Brazil; 6 Centro de Parasitologia e Micologia, Instituto Adolfo Lutz, São Paulo, Brazil

**Keywords:** *Schistosoma mansoni*, Human tumor necrosis factor alpha, Egg-laying, Glucose metabolism, Cell signaling, Host–parasite interaction

## Abstract

Several studies have described the effects of human tumor necrosis factor alpha (hTNF-α) on *Schistosoma mansoni*. hTNF-α affects the worm’s development, metabolism, egg-laying, gene expression and protein phosphorylation. The available data on the influence of hTNF-α on egg-laying in *S. mansoni* are controversial, but understanding the mechanism of egg-laying regulation in this species is essential in combating schistosomiasis. We characterized the effects of in vitro treatment of *S. mansoni* adult worms with different doses of hTNF-α (5, 20 and 40 ng/ml) for 5 days. We explored the effects on egg-laying rate, glucose levels, ATP metabolism, and messenger RNA (mRNA) expression levels of lactate dehydrogenase, glucose transporters and the parasite gene which acts as an hTNF-α receptor, *Sm*TNFR. hTNF-α influenced egg-laying in a time- and dose-dependent manner: at a dose of 40 ng/ml, egg-laying increased on day 2 and decreased on days 3 and 4; at 20 ng/ml, egg-laying decreased on day 3; while at 5 ng/ml, egg-laying decreased on day 4. The total number of eggs produced was not affected by the different treatments, but the egg-laying dynamics were: the median egg-laying time decreased significantly with treatment, and egg developmental stages and size were also affected. At 5 and 20 ng/ml hTNF-α, lactate production diminished on day 3 up to day 5, while glucose uptake increased on day 5. At 40 ng/ml, glucose uptake diminished on day 1 up to day 3, while ATP accumulation was detected on day 5. No significant changes in mRNA expression were detected in any of the treatments. We found that crosstalk involving hTNF-α and parasite signaling plays a role in the fine-scale regulation of the worm’s metabolism and physiology, and points to new strategies for disease control.

**Graphical abstract:**

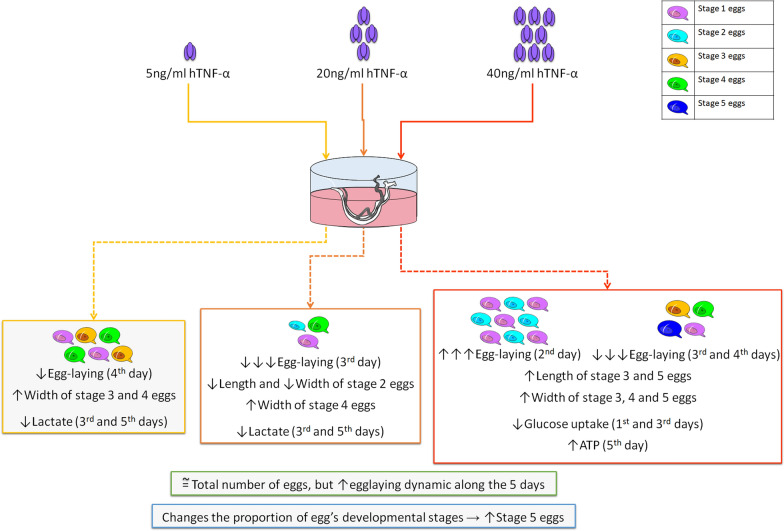

**Supplementary Information:**

The online version contains supplementary material available at 10.1186/s13071-022-05278-8.

*Schistosoma mansoni* is the major causative agent of schistosomiasis, which affects more than 200 million people worldwide, especially in developing countries. Schistosomiasis is a debilitating disease due to the parasite’s egg-laying and the subsequent formation of periovular granulomas, which are the key events in the pathogenesis of this disease [1].

An understanding of the host–parasite interactions in schistosomiasis is important as it increases our knowledge of the biology of schistosomes and is necessary for the development of new strategies against this disease. Factors that are important for the parasite’s development, and the signaling elements responsible for molecular crosstalk between the parasite and the host, are key topics for further exploration [2]. Tumor necrosis factor alpha (TNF-α) is an early pro-inflammatory cytokine produced in mammalians [3]. Several effects of human TNF-α (hTNF-α) on schistosomes have been described in the literature. Amiri et al. [4] found that, in schistosome-infected severe combined immunodeficiency (SCID) mice, hTNF-α increased egg-laying and restored granuloma formation. Controversially, Haseeb et al. [5] reported that in vitro treatment of *S. mansoni* with this human cytokine decreased egg-laying and increased tyrosine uptake in females. Similarly, Haseeb et al. [6] observed that males treated with hTNF-α increased their uptake of tyrosine. In 1999, Cheever et al. [7] found that egg-laying was delayed but normal in SCID immunodeficient mice (which produced less TNF-α due to the absence of B and T cells). A few years later, Davies et al. [8] reported the involvement of TNF-α in limiting liver disease and in helping the parasite to survive. Oliveira et al. [9] documented that hTNF-α induced changes in the expression profile of *S. mansoni* schistosomula and adult worms in vitro; later, Oliveira et al. [10] reported changes in the phosphorylation profile of male adult worms as a consequence of treatment with hTNF-α. Recently, Haseeb et al. [11] showed a reduction in methionine uptake and egg-laying in female worms treated in vitro with hTNF-α. Over the years, the effects of hTNF-α on *S. mansoni* have been shown to be both complex and interesting, although they remain controversial and difficult to understand.

In the present work, we aimed to study the in vitro effects of hTNF-α on *S. mansoni* glucose metabolism, gene expression and egg-laying regulation. For this purpose, *Mesocricetus auratus* were infected with 300 cercariae of *S. mansoni* (BH strain) through subcutaneous injection, then were euthanized and subjected to portal perfusion 7–8 weeks post-infection. Adult worms were recovered from the portal vein through perfusion with Roswell Park Memorial Institute medium (RPMI) medium with heparin (250 UI/l). The worms were then washed in RPMI and incubated in RPMI containing 10% bovine fetal serum with 1× antibiotic/antimycotic and gentamicin (50 mg/l) at 37 °C under 5% CO_2_. Different concentrations of recombinant hTNF-α (5, 20, 40 ng/ml; Sigma-Aldrich) were added to the culture medium; in parallel, the vehicle (Tris 10 mM pH 8.0) was added as the negative control. At least three biological replicates were performed for each parameter that was evaluated.

As a control of the effectiveness of our hTNF-α preparation, the activity of reconstituted hTNF-α was evaluated with a standard protocol, an apoptosis induction assay using a human epithelial type 2 (HEp-2) cell line. The cells were cultured in Eagle’s minimal essential medium containing 10% bovine fetal serum with 1× antibiotic/antimycotic (Gibco) and gentamicin (50 mg/l) at 37 °C under 5% CO_2_ in a culture plate with a coverslip on the bottom. After two days of culture, at 90% cell confluence, the cells were treated with reconstituted hTNF-α (20 ng/ml) in parallel with the respective negative control (treated with the same volume of Tris 10 mM pH 8 — the vehicle) for 18 h. After treatment, the coverslips with the attached HEp-2 cells were immersed in 100% ethanol for 2 h for fixation. Colorimetric immunohistochemistry of HEp-2 cells was performed with rabbit anti-cleaved caspase 3 antibody (PC679 Sigma-Aldrich) diluted to 1:100 and anti-mouse immunoglobulin G diluted to 1:1000, in accordance with the procedure described by Santos et al. [12]. Apoptotic cells were labeled with a brown dye and were counted in sets of 100 cells in both hTNF-α-treated and control samples (three biological replicates). Student’s *t*-test was used to compare the differences between the average number of apoptotic cells in the treated and control samples. The results are shown in Additional file 1: Fig. S1.

Treatment with reconstituted hTNF-α induced apoptosis in a significantly higher number of treated cells when compared with the control. This finding indicates that the activity of reconstituted hTNF-α was as expected in these experiments. The reconstituted cytokine was able to induce apoptosis in HEp-2 cells, which was expected, since Goodkin et al. [13] reported that hTNF-α induces apoptosis in these cells via caspase activation.

We evaluated the egg-laying of paired adult* S. mansoni* worms for 5 days at different concentrations of hTNF-α. Each pair was placed in a well of a 24-well plate, and eggs were counted every day for 5 days. We performed three independent experiments using a total of 27 adult pairs for each condition. The counting of eggs was performed using a blind assay (the egg counter was blind as to the treatment condition in each well). On the third day, the medium containing hTNF-α (and controls) was replaced.

Data analysis was performed with GraphPad Prism 7.03 (RStudio 2020). For the egg-laying, lactate production, glucose production, and reverse transcription–quantitative polymerase chain reaction (RT-qPCR) experiments, Shapiro–Wilk normality tests were performed using the data from all the groups to evaluate the normality of the distributions. For data with normal distributions, ANOVA [14] was performed for four different conditions, and Tukey’s test [15] was used to compare  two conditions. For data that were not normally distributed, the Kruskal–Wallis test [16] was used to make multi-comparisons, and the Wilcoxon pairwise test [17] was used to compare two conditions. The Benjamini–Hochberg correction [18] was used to adjust the *P*-values. Adjusted *P*-values < 0.05 were considered significant. All detailed information about the statistical analysis of each experiment and comparison is given in the supplementary tables together with the respective raw data.

The results are shown in Fig. 1. We observed a significant increase in egg-laying on day 2 after treatment with 40 ng/ml hTNF-α, a significant decrease in oviposition on day 3 after treatment with 20 and 40 ng/ml hTNF-α, and a significant decrease in egg-laying on day 4 after treatment with 5 and 40 ng/ml hTNF-α (Fig. 1a). When the total number of eggs produced across the 5 days was computed for each treatment condition, no significant difference in total egg output was observed between the treatments and the control (Fig. 1b). Additional file 2: Table S1 contains all the raw data of the egg-laying experiments and the results of the statistical analyses. Our results allowed us to calculate the egg-laying decay time using the Weibull distribution [19] to evaluate the effect of hTNF-α on the scale parameter (η) of egg-laying time of each group. The η of each group was compared by the Kruskal–Wallis test [16] (Additional file 2: Table S1, sheet C). This analysis showed a significant decrease in the half-life of egg-laying with the increase of the hTNF-α dose compared to the negative control (Fig. 1c).

**Fig. 1:**
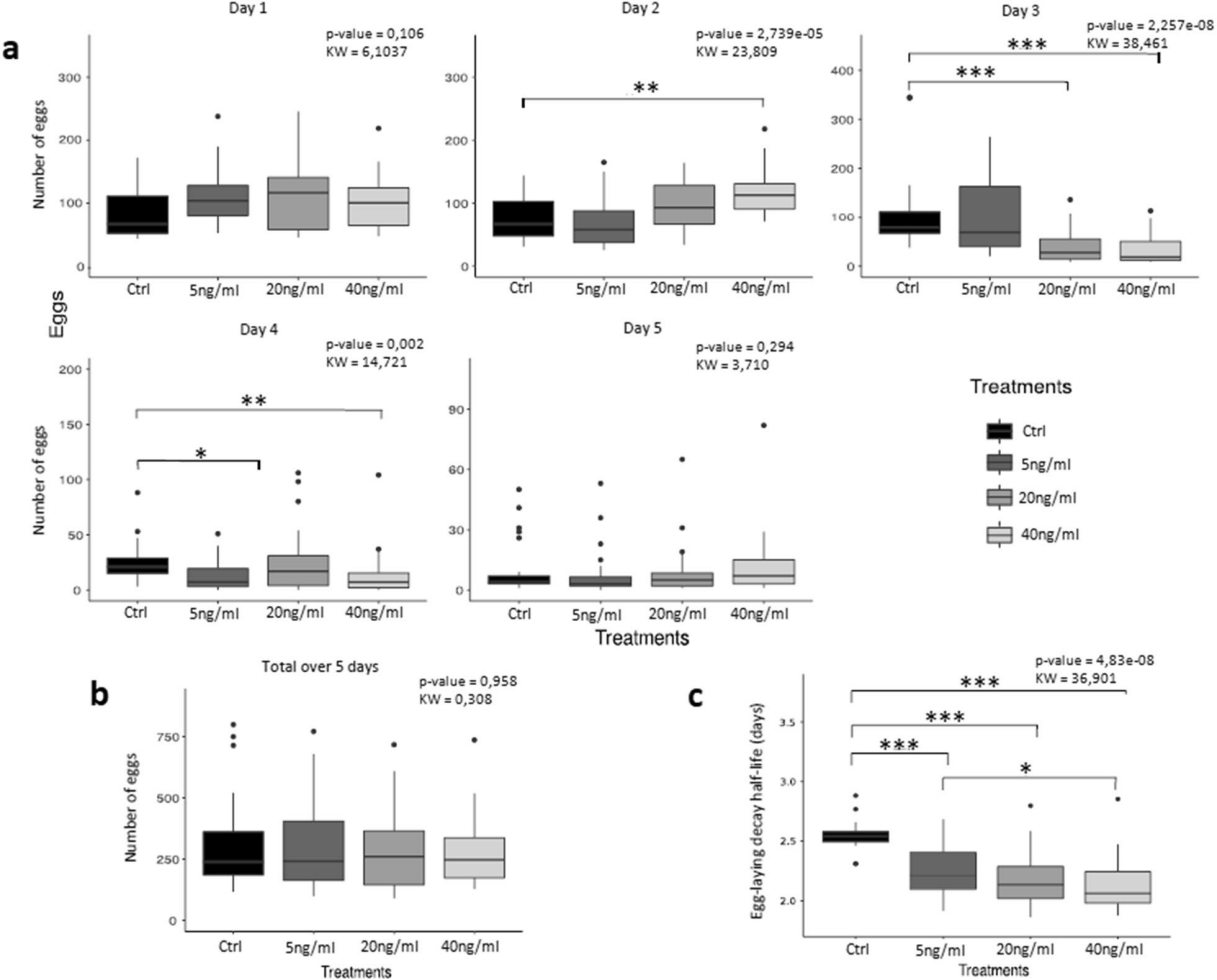
Effect of human tumor necrosis factor alpha (*hTNF*-*α*) on egg-laying in *Schistosoma mansoni*. **a** Box plots showing the number of eggs laid by paired worms treated with hTNF-α (at 5, 20 and 40 ng/ml) and the respective control (*Ctrl*) on each of the 5 days of experimentation. **b** Total number of eggs laid over the 5-day treatment period for each treatment condition. **c** Box plots of egg-laying half-life (days) for each treatment condition. Outliers are shown by* dots*. * P < 0.05, ** *P* < 0.001, *** P < 0.0001

Over the five days of the experiment, treatment with hTNF-α increased the egg-laying dynamic at all doses in a dose-dependent manner (the higher the treatment dose the shorter the egg-laying time).

We compared the proportions of the different developmental stages of the eggs among the control and hTNF-α-treated worms at the end of the five days of treatment. For this purpose, we treated adult pairs for five days, as previously described, using a Transwell in each well to retain the eggs when the medium was replaced on the third day. A chi-square test was used to determine if the differences were statistically significant. Additional file 3: Fig. S2 shows representative images of egg morphology for all five developmental stages in accordance with Cunha et al. [19] and egg classification.

There was a significant difference between all the treatment conditions and the control concerning the proportions of eggs at the different developmental stages (Fig. 2a). There was a higher proportion of eggs at stages 1 and 5 in the treated samples compared to the control. This result was expected because the treated parasites laid eggs faster than the control worms, and consequently, more eggs developed to the last stage in the treatments. The higher proportion of eggs at stage 1 may have been related to the fact that, as eggs were laid faster in the treated worms than in the controls, some of those in the former would not have had time to complete their development and would still have been at stage 1 on day 5 since egg-laying dramatically diminished between the fourth and the fifth day of the experiment. From these results, we can conclude that hTNF-α affects the process of egg maturation, which affects the parasite’s development. The raw data of the egg developmental stages are shown in Additional file 4: Table S2, sheet A.

**Fig. 2:**
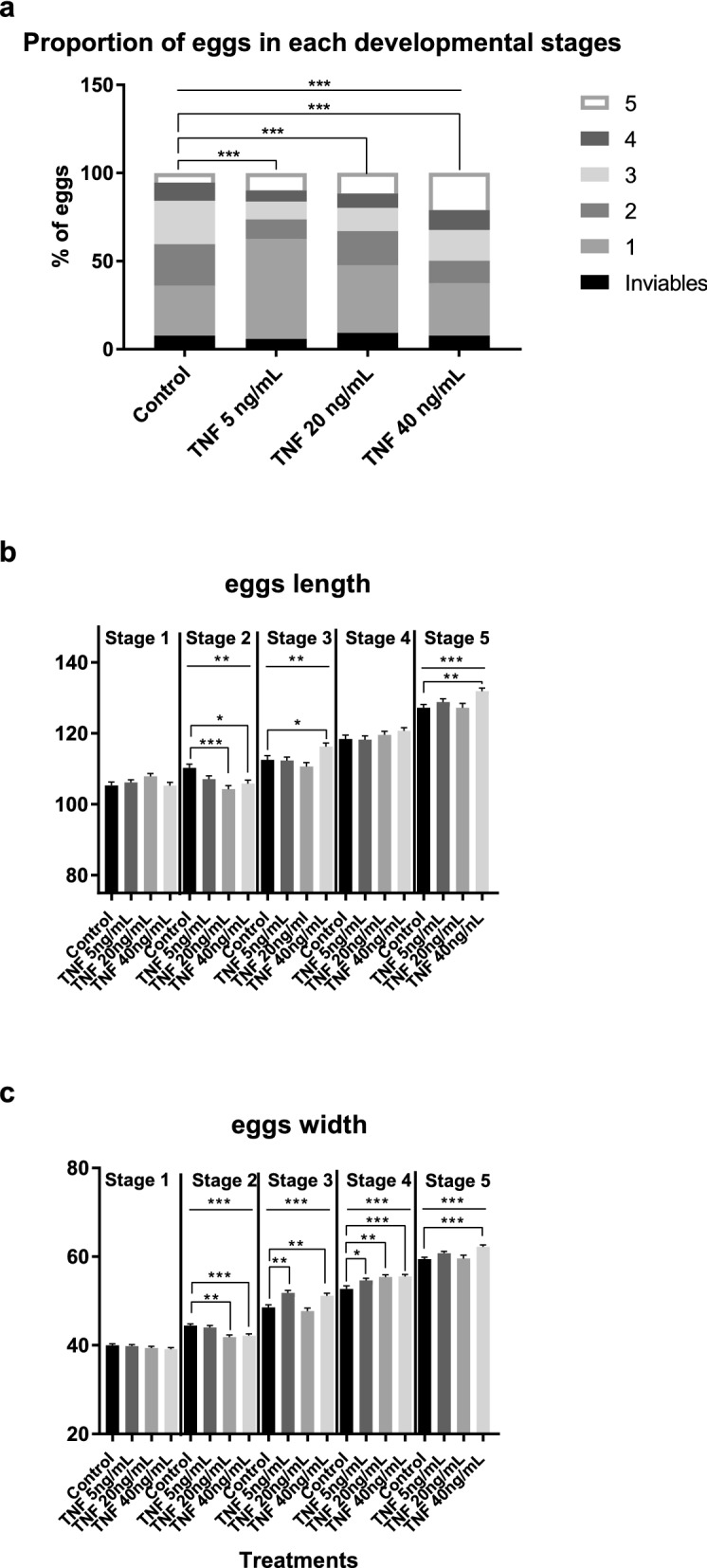
Effect of hTNF-α on the developmental stages and dimensions of *Schistosoma mansoni* eggs. **a** The proportion of eggs at each developmental stage for the control and hTNF-α-treated adult pairs after 5 days of treatment. Eggs were classified as inviable or at stage 1, 2, 3, 4, or 5 according to the morphological development of the embryo/miracidium. The chi-square test was applied among all conditions (*P* < 0.0001) and between each treatment condition vs the negative control (*** P < 0.001). **b** Mean ± SEM of egg length (*y*-axis; µm) at each developmental stage for the treatment groups. **c** Mean ± SEM of egg width (*y*-axis; µm) at each developmental stage for the treatment groups. The Kruskal–Wallis test (**a**) or ANOVA (**b**) was applied. * *P* < 0.01, ** *P* < 0.001, *** *P* < 0.0001

We measured the length and width of the eggs produced by the hTNF-α-treated worm pairs at each developmental stage, for each experimental condition, for comparison. A similar statistical approach was used to that in the egg count experiment to evaluate egg size (length and width). Additional file 4: Table S2, sheet B shows the results of the statistical analysis. In the hTNF-α treatments, the eggs at developmental stage 2 were smaller than those in the control, whereas those at stages 3, 4 (except for length) and 5 were bigger than those of the control. We suggest that hTNF-α may influence the parasite’s development. It is of interest that the eggs of the treated worms were bigger, as this suggests that hTNF-α may promote the development of the miracidia. In support of this, Liu et al. [20] reported that microRNA released in vesicles secreted by *Schistosoma japonicum* regulated host macrophage expression of TNF-α, which can be used by the parasite as a strategy to control the host immune response and its own development. Additional file 4: Table S2, sheets C and D document all the length and width data for all the treatment conditions, and the statistical analysis.

We measured the concentrations of lactate, glucose, and adenosine triphosphate (ATP) in the culture media of the adult pairs that were treated with hTNF-α, and in the respective negative control, at days 1, 3 and 5. We used two approaches for the measurement of these metabolites on day 5: replacement of the medium on day 3, or no replacement of the medium during the 5-day experiment. Five *S. mansoni* adult worm pairs were incubated in 1.5 mL of medium with different concentrations of hTNF-α and incubation times, as described above. The culture medium was centrifuged, and the supernatant was sent to the Central Laboratory of São Paulo Hospital (Universidade Federal de São Paulo; UNIFESP). Lactate and glucose were measured using a Cobas 6000 c501 analyzer with the Lactate Gem.2 kit and Glucose HK Gen.3 kit (Roche), respectively.

Figure 3 summarizes the results. There was a significant decrease in lactate levels in the medium in the treatments with 5 and 20 ng/ml hTNF-α on days 3 and 5 (where the medium had not been changed). An increase in lactate concentration was observed on day 5 in the treatment with 40 ng/ml hTNF-α; this increase was statistically significant when the medium had been changed on day 3.

**Fig. 3:**
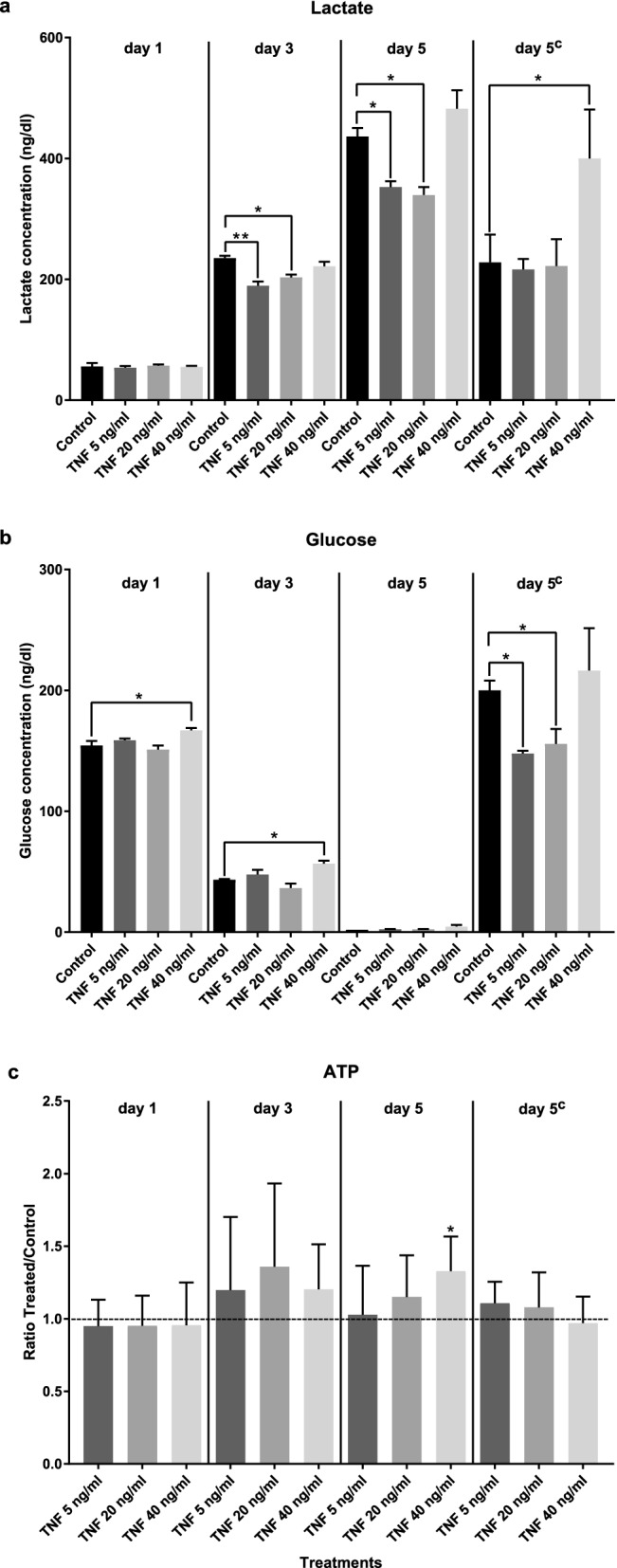
Concentrations of lactate (**a**), glucose (**b**) in the culture media and adenosine triphosphate (*ATP*) (**c**) in *Schistosoma mansoni* adult worms treated with hTNF-α (5, 20 and 40 ng/ml) on days 1, 3, 5 and 5^c^ (the superscript indicates that the medium had been replaced on day 3). Means are plotted and SEM are indicated by* vertical lines*. * *P* < 0.05, ** P < 0.005.** c** The *y*-axis indicates the ratio of the luminescence values of the treated/control worms

Glucose uptake in the 40 ng/ml hTNF-α treatment decreased significantly on days 1 and 3 (higher quantities of glucose remained in the treated medium compared with the control medium). When the medium was replaced on day 3, an increase in glucose uptake was observed on the day 5 for worms treated with 5 and 20 ng/ml hTNF-α. Additional file 5: Fig. S3A, B shows the absolute mean concentrations of lactate and glucose that were measured in the media. An increase in lactate production and a decrease in glucose were observed during the culture. Extremely low amounts of glucose remained on day 5 when the medium had not been changed, but when the medium had been replaced on the day 3 (as performed in the egg-laying experiments), a reasonable amount of glucose remained in the media on day 5.

Since one subunit of ATP synthase was differentially phosphorylated upon treatment of adult *S. mansoni* with hTNF-α [10], and lactate and glucose concentrations were affected by the treatment of adult pairs with hTNF-α, we evaluated ATP accumulation in the worms treated with hTNF-α. For this experiment, five adult worm pairs were incubated in 5 ml of media with different concentrations of hTNF-α and the respective negative control, as described above, for 1, 3 and 5 days. After the incubation, the worm pairs were transferred to microtubes containing 200 µl of RPMI, macerated with a pestle for 5 min and centrifuged at full speed for 5 min. Then 100 µl of the supernatant was pipetted into a white 96-well plate with 100 µl of CellTiter-Glo reagent (Promega). The plate was mixed for 2 min and luminesce was determined using an Enspire Multimode Plate Reader (PerkinElmer). Due to the variability in the detected luminescence in each experimental batch and/or replica, and the arbitrary unit of measured luminescence, we calculated the ratio of the luminescence values of treated/control worms to achieve normalization among the different experiments (as an indirect estimation of the amounts of ATP). A one-sample *t*-test [21] with a theoretical mean of 1.0 (control) was performed to evaluate the differences between the averages of the treated/control ratio of worms in the ATP assay.

We observed a significant increase in ATP levels on day 5 (where the medium had not been changed) when worms had been treated with 40 ng/ml hTNF-α (Fig. 3c). The luminescence values are shown in Additional file 5: Fig. S3c. Additional file 6: Table S3 contains all the raw data of lactate, glucose, and ATP quantitation, along with the statistical analysis.

We investigated the mRNA expression levels of *S. mansoni* tumor necrosis factor receptor (*Sm*TNFR), lactate dehydrogenase (LDH), and the glucose transporter genes for all hTNF-α treatment conditions using a qPCR approach. We aimed to validate the results reported in Oliveira et al. [9], i.e. upregulation of LDH expression upon treatment with 20 ng/ml hTNF-α for 24 h, and to possibly explain the changes in lactate and glucose concentrations observed here.

Total RNA from hTNF-α-treated and control adult pairs was extracted using RNeasy Micro Kits (QIAGEN). Samples were quantified using a fluorometer (Qubit; Applied Biosystems), and RNA quality was evaluated via microfluidic electrophoresis using a Bioanalyzer (Agilent Technologies) according to the manufacturer’s instructions. Complementary DNA was synthesized with 200 ng of RNA per sample using SuperScript IV Reverse Transcriptase (Thermo Fisher Scientific). Real-time PCR was performed using Sybr Green reagent (Roche), and gene expression levels of LDH (Smp_033040), *Sm*TNFR (Smp_168070) and glucose transporters, *S. mansoni* glucose transporter protein 1 (SGTP1) (Smp_012440), SGTP2 (Smp_046790), SGTP3 (Smp_127200) and SGTP4 (Smp_103410), were measured. The primer efficiencies were tested, and all of them were between 90 and 110%. Smp_090920 and Smp_062630 were used as reference genes [22]. The primer sequences are shown in Additional file 4: Table S3. Relative gene expression was calculated according to Taylor et al. [23]. All the RT-qPCR assays were performed according to the Minimum Information for Publication of Quantitative Real-Time PCR Experiment guidelines [24].

We verified that no significant changes in the mRNA expression levels of the investigated genes occurred under any treatment condition. Although mean LDH expression increased with treatment of 20 ng/ml of hTNF-α, in accordance with Oliveira et al. [9], the result was not statistically significant (data not shown). We did not observe any other significant differences in the expression of SGTPs, which suggested that regulation at the protein level or post-translational mechanisms may play a role in the parasite’s signaling in response to hTNF-α.

As characterization of the effects of hTNF-α on the physiology and metabolism of *S. mansoni* is important for understanding the molecular basis of host–parasite interactions and platyhelminth biology, it may also point to new targets for the development of strategies against schistosomiasis. Bertevello et al. [25] recent description of 29 parasitic platyhelminth genes homologous to *Sm*TNFR is of particular relevance to this.

There is no consensus in the literature regarding the effect of hTNF-α on *S. mansoni* egg-laying. However, egg-laying is a central event for the pathogenesis and spread of schistosomiasis. Amiri et al. [4] found that the in vitro treatment of females with different doses of hTNF-α (10, 20 and 40 ng/ml) proportionally increased the number of eggs laid over 3 days. Haseeb et al. [5] showed that the in vitro treatment of females with 20, 40 and 100 ng/ml of hTNF-α induced a significant decrease in the number of eggs laid. Additionally, Cheever et al. [7] showed in vivo that SCID mice had fewer eggs in their tissues at the early infection stage than BALB/c mice; however, after 9 weeks of infection, no difference was observed between the two types of mice.

Here, we used paired adults in vitro to maintain an adequate assay environment, since the presence of male parasites acts as a signal for female worms to access key nutrients [26]. The median egg output per pair in all treatment conditions was higher on the first day, although there were no significant differences when compared to the control; on the second day of treatment, we observed a significant increase at 40 ng/ml hTNF-α. On the third day, we observed a significant decrease in the median egg output per pair for the treatments with 20 and 40 ng/ml hTNF-α, which corroborates Haseeb et al.’s [5] findings but contradicts those of Amiri et al. [4]. The most important observation was that, over the five days of treatment, the total number of eggs was not significantly different between the treatments and the control, which means that although the dynamics of egg-laying were affected, fecundity was not. The presence of host TNF-α made the pairs lay eggs faster (Fig. 1c), but not in higher or lower quantities (Fig. 1b); consequently a higher proportion of eggs developed to stage 5 and became bigger than those of the control. In general, our results can be interpreted in a similar way to those described by Cheever et al. [7], in which a delay in egg-laying was observed in in vivo experiments with SCID mice. It is apparent that hTNF-α accelerates egg-laying and helps in the development of the parasites, while at lower doses it delays oviposition.

To understand which mechanisms are involved in the regulation of egg-laying by hTNF-α, we measured glucose and lactate levels and examined whether these correlated with the expression of related genes. We did observe that when the lactate or glucose concentration changed significantly, there was also a significant change in egg-laying. On day 3 of treatment, when lactate production or glucose uptake was diminished, egg-laying decreased, indicating that metabolism plays a role in this and that it is regulated by hTNF-α, which thus interferes with the dynamics of egg production.

Egg production was limited in an ex vivo environment due to the exhaustion of metabolic resources (glycogen and fat) [26], and glucose is most probably directly used in worm metabolism [26]. Ex vivo/in vitro systems have several limitations, and new approaches to improve the in vitro culture of schistosomes have been developed [27]. Here we chose to use a traditional in vitro culture system to compare our results with those previously described in the literature [5, 11]. The effect of hTNF-α on glucose uptake by pairs of *S. mansoni* was subtle and not related to the mRNA expression of SGTPs. The changes in lactate production were not related to the expression level of LDH mRNA; the regulation of this enzyme is probably at the protein expression level and/or is a post-translational modification (as suggested by Oliveira et al. [10]). The observed increase in ATP on day 5 could be explained by the compromised rate of egg-laying at this time. The high demand for ATP was for oogenesis, and when ATP is not needed for this, it may accumulate.

We conclude that hTNF-α did not affect the fecundity of *S. mansoni* but did affect the dynamics of its egg production and subsequently the maturation and size of its eggs (which increased in the presence of this human cytokine); lactate production and glucose uptake may be of relevance to the regulation of egg-laying. Here, we demonstrated that molecular crosstalk between the host and the parasite plays a role in the fine regulation of platyhelminth physiology, and is important for the successful adaptation of this ecological relationship. Most parasitic platyhelminth species have homologous genes for *Sm*TNFR but not for hTNF-α [25], and may have established a homologous relationship with their hosts through their coevolution.

## Supplementary Information

**Additional file 1: Figure S1.** Apoptosis induction assay using HEp-2 cells with reconstituted hTNF-α.

**Additional file 2: Table S1.** Total egg counts in all experiments of treatment with hTNF-α (sheet A), respective statistical analysis (sheet B) and coefficient values of the factors tested using the Weibull distribution for total egg counts (sheet C).

**Additional file 3: Figure S2.** Egg developmental stages after treatment with hTNF-α.** a** Oogram of the intestinal mucosa of a *Schistosoma mansoni*-infected hamster with inviable eggs, eggs at stage 1 (a central group of germ cells), stages 2 and 3 (the group of germ cells has increased in size, in length and width, and expanded in all directions towards the eggshell), stage 4 (the embryo’s structure can be observed) and stage 5 (miracidia are completely formed).** b** Representation of egg classification for the in vitro experiment (egg stages are indicated).

**Additional file 4: Table S2.** Distribution of egg developmental stages according to treatment condition (sheet A), respective statistical analysis (sheet B), length (sheet C) and width data (sheet D) and statistical analysis (sheet E).

**Additional file 5: Figure S3.** Mean (± SD) of absolute values of lactate (**a**), glucose (**b**) in the medium and ATP (**c**) in the *S. mansoni* adult worms treated with hTNF-α measured on days 1, 3 and 5.

**Additional file 6: Table S3.** Raw data and statistical analysis of lactate (sheets A and B), glucose (sheets C and D) and ATP levels (sheets E and F) of  *S. mansoni* adult worms treated with hTNF-α.

**Additional file 7: Table S4.** Primer sequences used in the RT-qPCR experiments.

## Data Availability

All data generated during this study are included in this published article and its additional files.

## Abbreviations

ATP: Adenosine triphosphate
HEp-2: Human epithelial type 2
hTNF-α: Human tumor necrosis factor alpha
LDH: Lactate dehydrogenase
RPMI: Roswell Park Memorial Institute medium
RT-qPCR: Reverse transcription–quantitative polymerase chain reaction
SCID: Severe combined immunodeficiency
SGTP: *Schistosoma mansoni* glucose transporter protein
*Sm*TNFR: *Schistosoma mansoni* tumor necrosis factor receptor
