# Supplementary material for: Human tumor necrosis factor alpha affects the egg-laying dynamics and glucose metabolism of Schistosoma mansoni adult worms in vitro
Source: Parasit Vectors. 2022 May 24;15:176. doi: 10.1186/s13071-022-05278-8 (PMC9128126; doi:10.1186/s13071-022-05278-8)
Supplement: Supplementary file 1 — Additional file 1: Figure S1. Apoptosis induction assay using HEp-2 cells with reconstituted hTNF-α. [file 13071_2022_5278_MOESM1_ESM.pdf]

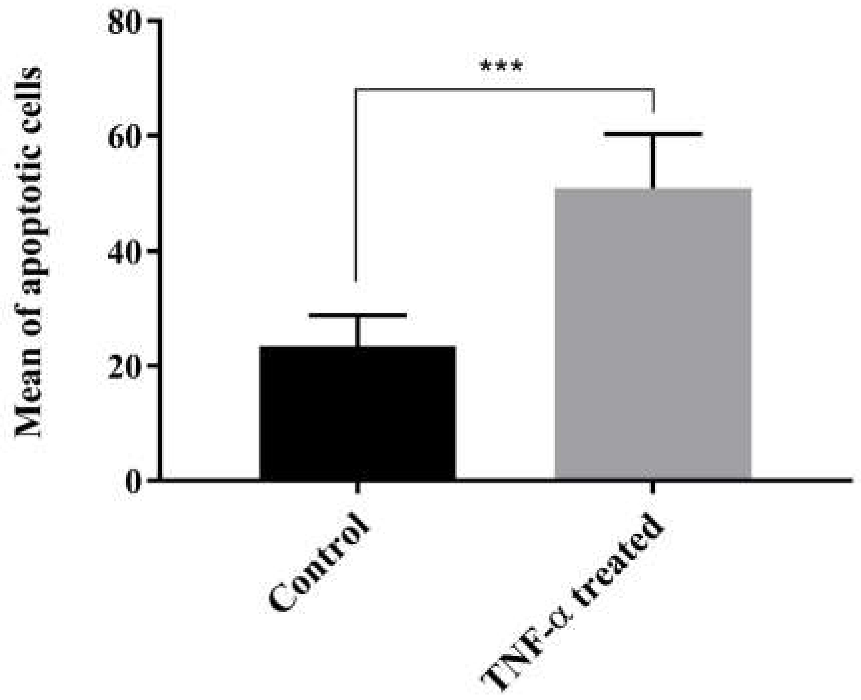

Figure S1. Apoptosis induction assay with the reconstituted hTNF-α. Means with SDs for apoptotic HEp-2 cells treated with 20ng/mL hTNF-α (grey bar) and the control (black bar, untreated). Student's (unpaired) t-test. *** $p<0.001$
