# Supplementary figures and images for: Human tumor necrosis factor alpha affects the egg-laying dynamics and glucose metabolism of Schistosoma mansoni adult worms in vitro
Source: Parasit Vectors. 2022 May 24;15:176. doi: 10.1186/s13071-022-05278-8 (PMC9128126; doi:10.1186/s13071-022-05278-8)

**a**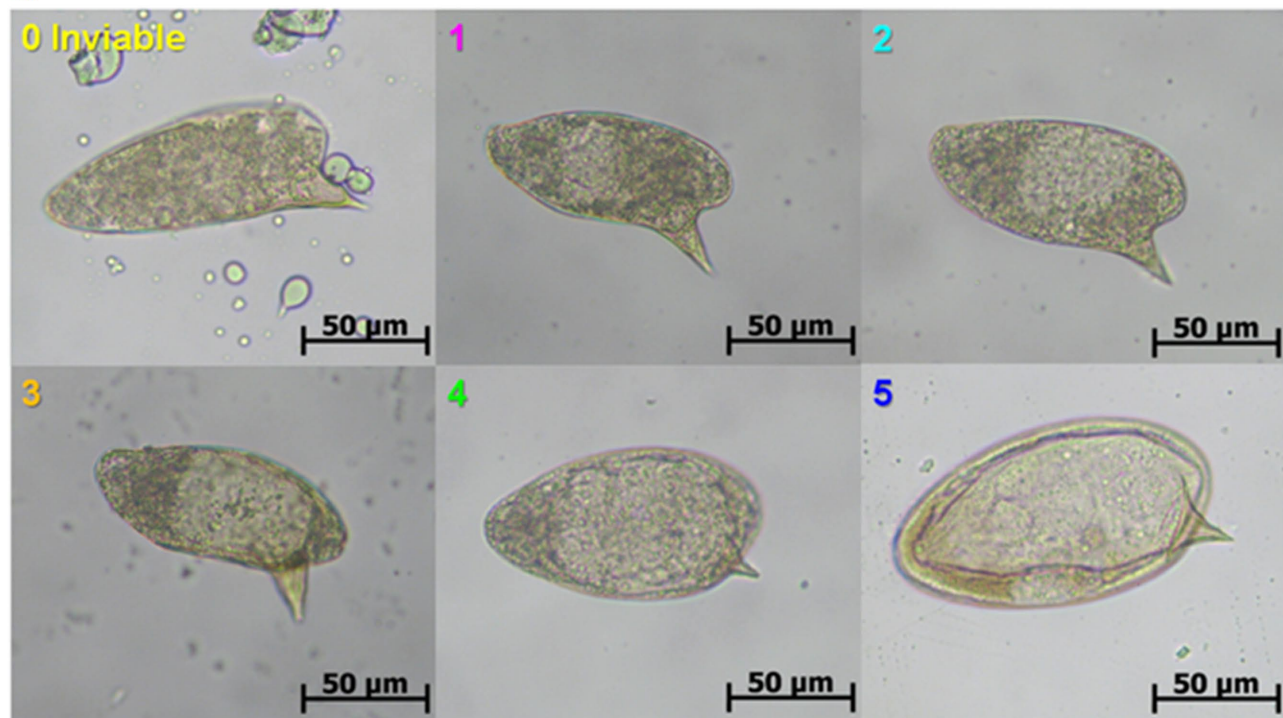**b**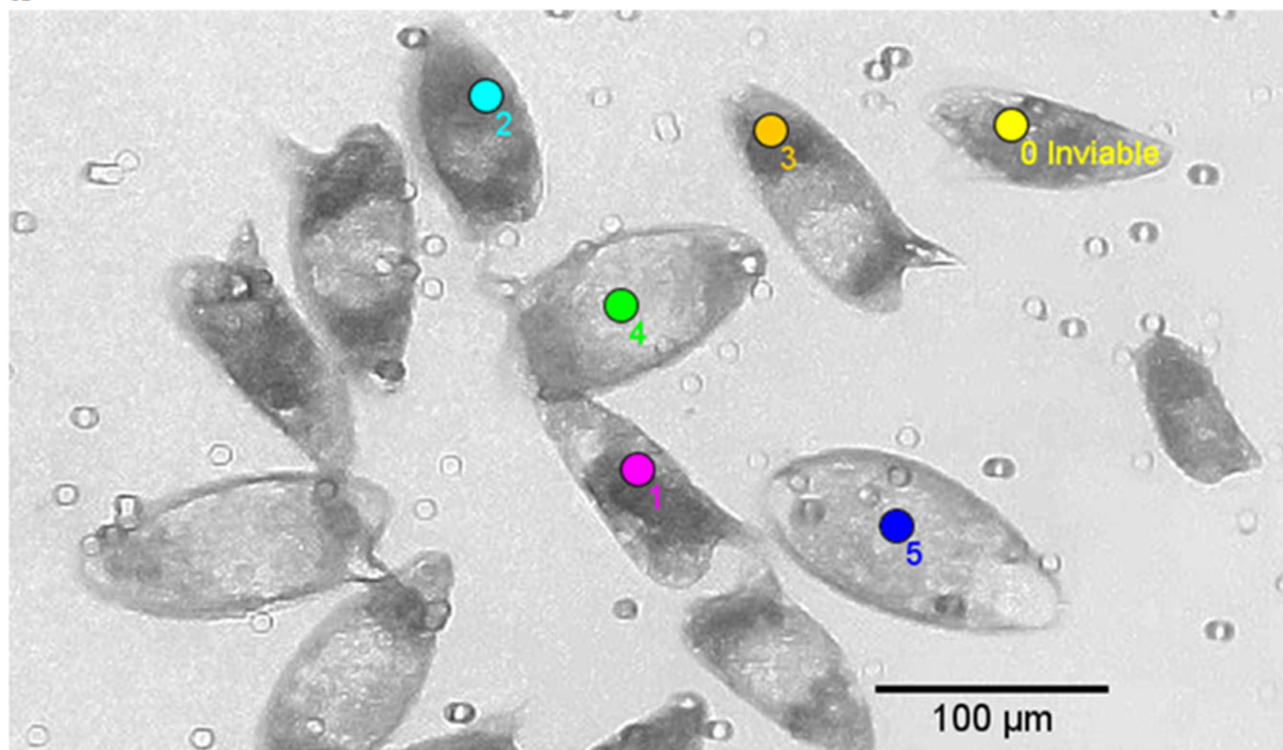

Supplement: Supplementary file 3 — Additional file 3: Figure S2. Egg developmental stages after treatment with hTNF-α. a Oogram of the intestinal mucosa of a Schistosoma mansoni-infected hamster with inviable eggs, eggs at stage 1 (a central group of germ cells), stages 2 and 3 (the group of germ cells has increased in size, in length and width, and expanded in all directions towards the eggshell), stage 4 (the embryo’s structure can be observed) and stage 5 (miracidia are completely formed). b Representation of egg classification for the in vitro experiment (egg stages are indicated). [file 13071_2022_5278_MOESM3_ESM.pdf]

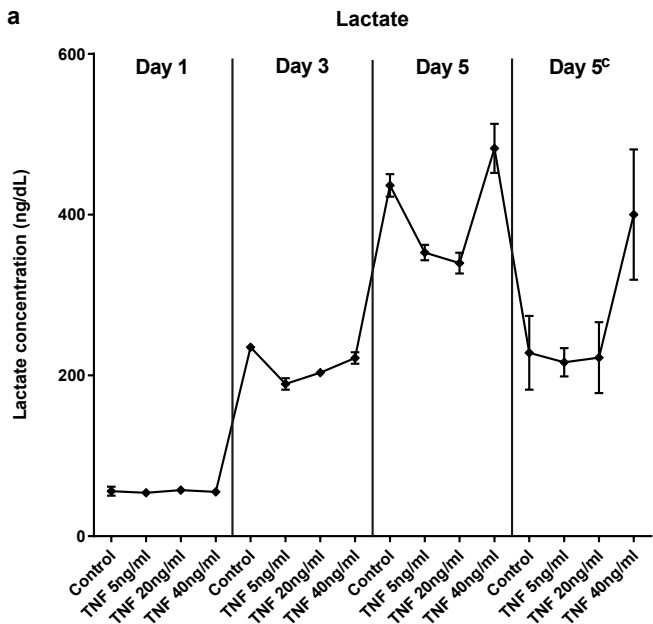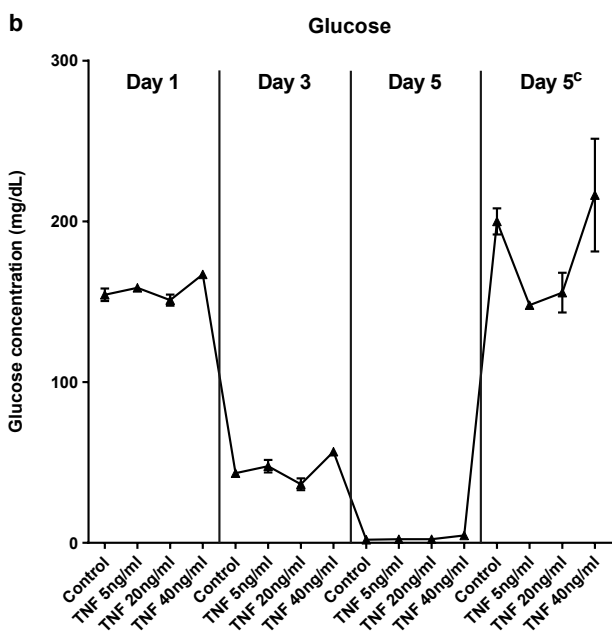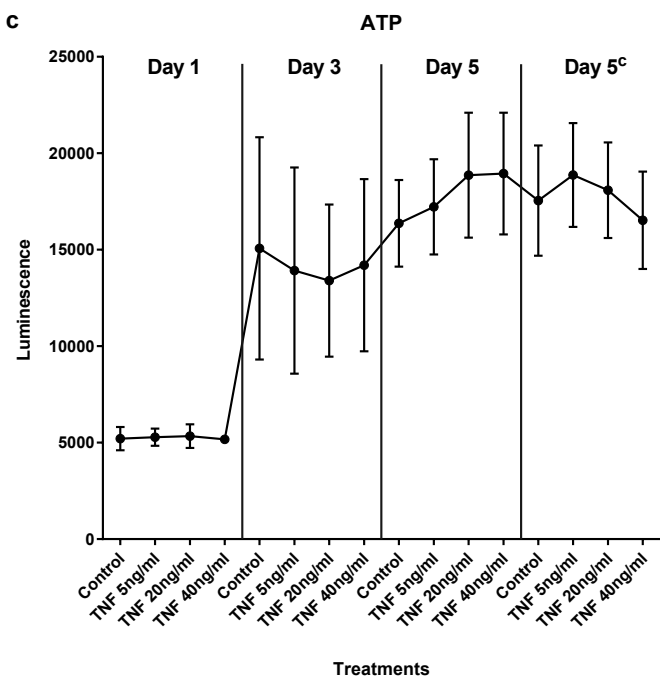

Supplement: Supplementary file 5 — Additional file 5: Figure S3. Mean (± SD) of absolute values of lactate (a), glucose (b) in the medium and ATP (c) in the S. mansoni adult worms treated with hTNF-α measured on days 1, 3 and 5. [file 13071_2022_5278_MOESM5_ESM.pdf]
